# Utilization of Jujube Biomass to Prepare Biochar by Pyrolysis and Activation: Characterization, Adsorption Characteristics, and Mechanisms for Nitrogen

**DOI:** 10.3390/ma13245594

**Published:** 2020-12-08

**Authors:** Di Zhang, Tongtong Wang, Jinhu Zhi, Qiangqing Zheng, Qiling Chen, Cong Zhang, Yalong Li

**Affiliations:** 1 College of Plant Sciences, Tarim University, Alar 843300, China; dizhang@nwafu.edu.cn (D.Z.); zc1569325100@163.com (C.Z.); 2 College of Natural Resources and Environment, Northwest A & F University, Yangling 712100, China; wangtongtong1993@nwafu.edu.cn; 3 Research Center of Oasis Agricultural Resources and Environment in Sourthern Xinjiang, Tarim University, Alar 843300, China; 4 Institute of Forestry and Horticulture, Xinjiang Academy of Agricultural Reclamation Sciences, Shihezi 832000, China; zhengqq369@163.com (Q.Z.); cql619@163.com (Q.C.); 5 Changjiang River Scientific Research Institute, Wuhan 430012, China; lyalong888@163.com

**Keywords:** jujube biochar, carbonization process, adsorption characteristics, nitrate and ammonium, mechanisms

## Abstract

The rapid advancement of jujube industry has produced a large amount of jujube biomass waste, requiring the development of new methods for utilization of jujube resources. Herein, medium-temperature pyrolysis is employed to produce carbon materials from jujube waste in an oxygen-free environment. Ten types of jujube biochar (JB) are prepared by modifying different pyrolysis parameters, followed by physical activation. The physicochemical properties of JB are systematically characterized, and the adsorption characteristics of JB for NO_3_^−^ and NH_4_^+^ are evaluated via batch adsorption experiments. Furthermore, the pyrolysis and adsorption mechanisms are discussed. The results indicate that the C content, pH, and specific surface area of JB increase with an increase in the pyrolysis temperature from 300 °C to 700 °C, whereas the O and N contents, yield, zeta potential, and total functional groups of JB decrease gradually. The pyrolysis temperature more significantly effects the biochar properties than pyrolysis time. JB affords the highest adsorption capacity for NO_3_^−^ (21.17 mg·g^−1^) and NH_4_^+^ (30.57 mg·g^−1^) at 600 °C in 2 h. The Langmuir and pseudo-second-order models suitably describe the isothermal and kinetic adsorption processes, respectively. The NO_3_^−^ and NH_4_^+^ adsorption mechanisms of JB may include surface adsorption, intraparticle diffusion, electrostatic interaction, and ion exchange. In addition, π–π interaction and surface complexation may also be involved in NH_4_^+^ adsorption. The pyrolysis mechanism comprises the combination of hemicellulose, cellulose, and lignin decomposition involving three stages. This study is expected to provide a theoretical and practical basis for the efficient utilization of jujube biomass to develop eco-friendly biochar and nitrogenous wastewater pollution prevention.

## 1. Introduction

The jujube (*Ziziphus jujuba* Mill., 1768) tree is widely distributed in southern and eastern Asia, Europe, Americas, and Australia [1], and can be a valuable resource of biomass energy owing to its high heating value and environmental resilience [2]. Currently, the global production of fresh jujube exceeds 10 million tons per year, and the demand of related products is consistently increasing each year because jujube can be used as food and for treating various diseases [1]. China has the largest planted area (>37,000 km^2^) and highest production (80% of the total world production) of jujube based on the data reported by the National Bureau of Statistics of China in 2017 [1,3]. However, a large amount of jujube branches are discarded during pruning and metabolisms due to the lack of innovative product development technologies, and the utilization of jujube biomass is very low in many areas [4,5]. Daoud et al. [6] investigated the jujube biomass in Algeria and identified it as a new agricultural waste that could provide abundant and low-cost biomaterial precursors. Sun et al. [7] argued that jujube wastes are difficult to decompose because of their complex textures. Therefore, development of new methods to enhance the comprehensive utilization of jujube biomass is necessary. In chemical industry, carbonized biomass can be used to obtain carbon materials with outstanding performances [8,9,10,11]. According to Yan et al. [12], the pyrolysis is also a frequently used and major carbonization process, and the basic physicochemical properties of the pyrolysis products may be improved by post-treatment, mainly involving physical and chemical activation processes. Biochar is a carbon-rich, stable, and highly aromatic solid product obtained from the pyrolysis of various biomass feedstocks under limited oxygen supply and in a specific temperature range (<700 °C) [13,14,15,16]. Furthermore, owing to the porous structure, large surface area, and abundant functional groups (i.e., free radicals and surface charges) of the biochar, it affords high adsorption capacity and stability [17,18,19,20]. Biochar has been widely used in agricultural and environmental protection fields such as electrochemical energy storage, pollution remediation, and preparation of modified materials for adsorption, catalysis, and soil improvement [18,19,20,21,22,23]. However, there is a lack of reports on using jujube as an ideal biomass precursor to prepare biochar.

Nitrogen is a key constituent of biological macromolecules such as proteins and nucleic acids, and it is one of the essential elements that promotes the growth and metabolic processes of living organisms (animals, plants, and microorganisms) [24]. Unfortunately, because of the excessive application of artificial fertilizer in agricultural production, nitrogen is not completely utilized and absorbed, causing non-point source pollution that in turn leads to water eutrophication and groundwater pollution [25]. This outcome not only endangers the health of humans and growth of plants and animals, but also decreases the quality of water resources and increases the cost of their remediation [26]. Biochar adsorption to remove excess nitrogen from various complex environments has been used for water treatment [27]. Nitrogen is present in the environment as nitrate (NO_3_^−^-N) and ammonium (NH_4_^+^-N) species, and therefore, these were selected as the target species in this study. Currently, the application of adsorption is a key research hotspot [28]. Alsewaileh et al. [29] reported that the biochar derived from date palm afforded a low NO_3_^−^ adsorption with an adsorption capacity of 0.12–8.37 mg·g^−1^ at different initial pH values, addition dosages, and pyrolysis temperatures. Cui et al. [30] studied the adsorption of NH_4_^+^ on six types of biochar prepared from wetland plants and found that the maximum adsorption capacity ranged from 2.12 mg·g^−1^ (*V. zizanioides* biochar) to 13.35 mg·g^−1^ (*C. indica* biochar). In addition, a few systematic studies have been performed for the utilization of jujube biomass, but many studies reporting the dynamic effects of micro-indexes lack an in-depth investigation [8]. In summary, jujube branches were used as a raw material for medium-temperature carbonization under oxygen-free conditions by controlling the pyrolysis temperature and time. Thereafter, 10 types of biochar were physically activated using ultrasound. Various characterization methods were used to systematically determine the effects of different pyrolysis parameters on the physicochemical properties of JB. The adsorption of NO_3_^−^ and NH_4_^+^ on JB was analyzed by batch adsorption experiments, and the pyrolysis and adsorption mechanisms were elucidated. 

## 2. Materials and Methods

### 2.1. Preparation of Biochar

**Figure 1:**
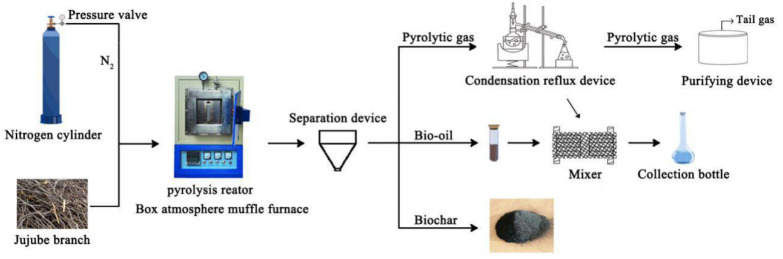
Schematic for carbonization process of jujube biomass.

The jujube branch feedstocks were collected from the Horticultural Experiment Station, Tarim University, Alar City, Xinjiang Uygur Autonomous Region, China. Then, a GF11Q-B box atmosphere muffle furnace (Nanjing Boyuntong Instrument Technology Co., Ltd., Nanjing, China) was used to perform pyrolysis at temperatures of 300, 400, 500, 600, and 700 °C for 1 and 2 h. The heating rate was set at 10 °C·min^−1^, and nitrogen gas was used to achieve the oxygen-free environment. Byproducts were collected using a condensing reflux device (Figure 1). Ultrasonication was performed in the KQ-300ES constant-temperature ultrasonic machine with numerical control (Kunshan Ultrasonic Instrument Co., Ltd., Kunshan, China). During ultrasonication, a specific amount of biochar was first weighed into a beaker, and 200 mL of deionized water was added for dispersion. An ultrasonication time of 30 min, frequency of 40 kHz, and power of 300 W were used. Finally, all solutions were filtered through a 0.45-μm filter membrane to recover JB, which was then dried at 105 °C for more than 8 h.

### 2.2. Characterization

The zeta potential was measured using a NanoZS90new zeta potential analyzer (Malvern Panalytical Ltd., Malvern, UK), and proximate analysis was performed using a XKGF-6000A automatic industrial analyzer (Henan Xinke Analytical Instruments Co., Ltd., Hebi, China). Detailed standard analytical methods of the element analysis, the scanning electron microscope (SEM), the Brunauer–Emmett–Teller (BET) specific surface area (S_BET_) and pore size analysis, the X-ray photoelectron spectroscopy (XPS), and the mineral species (X-ray diffraction, XRD), and the Fourier Transform Infrared (FTIR) were displayed in the supplementary materials. Thermogravimetric (TG) analysis was performed using standard method by a TGA2 analyzer (Mettler Toledo International Trading Co., Shanghai, China).

### 2.3. Batch Experiments

Standard NO_3_^−^ and NH_4_^+^ solutions were prepared by dissolving different amounts of KNO_3_ and NH_4_Cl, respectively, in deionized water. Then, 0.1000 g of JB sample was accurately weighed and immersed into 50 mL of NO_3_^−^ or NH_4_^+^ solution in a 250-mL conical flask, and 0.01 mol·L^−1^ KCl was used as a background electrolyte by adjusting the pH of the solution to 7–8. The flask covered with a plastic film was shaken at 150 rpm for 3 h in an air bath oscillator at a constant temperature 25 °C (±1 °C). The mixture was filtered using a 0.45-μm microfiltration membrane to obtain the filtrate, which was analyzed using a 3-AA3 continuous flow auto analyzer (Bran + Luebbe, Delavan, WI, USA) to determine the equilibrium concentration. All treated samples and blanks (H_2_O and JB) were analyzed in triplicate and the mean values were calculated by statistical methods. The adsorption capacity (*Q_e_*, mg·g^−1^) and removal rate (*R_e_*, %) at equilibrium were calculated as

(1) $$Q_{e}=(C_{0}-C_{e})\frac{V}{m}$$

(2) $$R_{e}=(1-\frac{C_{e}}{C_{0}})\times 100\%$$

where *C*_0_ and *C_e_* are the initial and equilibrium concentrations (mg·L^−1^), respectively. *m* is the mass of added JB (g), and *V* is the solution volume (L). The detailed operation of batch experiment is described below. (1) Isothermal adsorption: the initial NO_3_^−^ and NH_4_^+^ concentrations were established at 1, 5, 10, 15, 20, 30, 40, 50, and 100 mg·L^−1^, respectively; (2) Adsorption kinetics: the adsorption times of NO_3_^−^ and NH_4_^+^ were established at 5, 10, 20, 30, 60, 180, 300, 600, 900, 1200, 1440, and 2880 min, respectively; (3) Effect of addition dosage on adsorption: the addition dosage of NO_3_^−^ and NH_4_^+^ were set as 0.02, 0.04, 0.08, 0.12, 0.16, 0.24, and 0.32 g, respectively. Other settings were the same as the batch experiment above.

### 2.4. Data Analysis

To better investigate the adsorption characteristics, the isotherm and kinetic adsorption models [31,32,33] were employed in Table 1. The detailed analysis method and the software used can be found in the supplementary materials. Using Gaussian propagation to calculate the uncertainty of elemental analysis parameters. More detailed information can be found on the website (https://en.wikipedia.org/wiki/Propagation_of_uncertainty). For example, the formula for “the uncertainty analysis of (*O*/*C*)” is

(3) $$S_{O/C}=\sqrt{{\left(\frac{1}{C}\right)}^{2}\times {S_{O}}^{2}+{\left(-\frac{O}{C^{2}}\right)}^{2}\times {S_{C}}^{2}}$$

where *C* and *O* are directly measured element content, and the uncertainty of (*O* + *N*)/*C* also be calculated by the above similar method.

Where *Q_e_* and *Q_t_* are the adsorbed capacity (mg·g^−1^) at an equilibrium concentration (*C_e_*, mg·g^−1^) and given time of NO_3_^−^ in solution, respectively. *Q_m_* (mg·g^−1^) denotes the maximum adsorption capacity. *a* and *K_F_* are the Langmuir (mg·L^−1^) and Freundlich (mg·g^−1^) constants, respectively. 1/*n* is the heterogeneity factor. *A* is the equilibrium binding constant (mg·L^−1^), and *B* is the Temkin constant related to the adsorption heat. *β* is the D-R model coefficient (mol^2^·J^−2^), *Q*_0_ is the maximum unit adsorption capacity (mmol·g^−1^), *ε* is the Polanyi adsorption potential, *R* is the ideal gas constant 8.314 J·(mol·K)^−1^, *T* is the absolute temperature, and *E* is the adsorption free energy (J·mol^−1^). *k*_1_, *k*_2_, and *k_i_* are the rate constants for the pseudo-first-order (min^−1^), pseudo-second-order (g·mg^−1^·min^−1^), and the intraparticle diffusion (mg·g^−1^·h^−1/2^) rate constant, respectively. *h* is the initial adsorption rate (mg·g^−1^·min^−1^). *a’* is the initial sorption ratio (g·mg^−1^·min^−1^), and *b* is the desorption constant (g·mg^−1^), *C* is a constant indicating the number of boundary layers of the adsorbent.

## 3. Results and Discussions

### 3.1. Biochar Characterization

#### 3.1.1. Basic Properties

The proximate analysis of jujube biomass are shown in Table 2 and the basic physicochemical properties of JB under different carbonization conditions are included in Table 3. Table 2 shows that the jujube biomass contains a large amount of volatile organic matter and fixed carbon, which can be used as an ideal material precursor. With an increase in the pyrolysis temperature from 300 °C to 700 °C, the C content, pH, S_BET_, and pore volume of JB increase, while the O and N contents, yield, zeta potential (its absolute value increase), and average pore size of JB gradually decrease (Table 3). The element content of H could be calculated indirectly and displayed in Table S1. As the pyrolysis temperature increases, the H element content in JB has no obvious change trend, but as the pyrolysis time increases, the H element content generally decreases. There are two important indicators in polymer elemental analysis: *O*/*C* can be used to determine the aromatization of the structure and compositions of high-molecular-weight polymers, while (*O* + *N*)/*C* can describe the polarity of the adsorbent [8,34]. As summarized in Table 3, the *O*/*C* value decreases significantly with an increase in the pyrolysis temperature, which is mainly due to the intense dehydrogenation and deoxygenation reactions at increased temperatures; this also indicates that an increase in temperature can promote the breakage of weak chemical bonds and formation of condensation products [35]. The (*O* + *N*)/*C* value decreases gradually with an increase in the pyrolysis temperature, indicating a decrease in the JB polarity, which is mainly indicated by the loss of oxygen-containing functional groups. The uncertainty analysis of *O*/*C* and (*O* + *N*)/*C* by Gaussian propagation showed a slight downward trend as the pyrolysis temperature increases both for pyrolisys time 1 h and 2 h, And these values are less than 0.5, which may mean that the indirect calculated values can be trusted [9]. As a result, the biochar prepared at high temperature is more stable. Furthermore, the physicochemical properties of JB also follow the above trend with an increase in the pyrolysis time. However, compared to the pyrolysis temperature, pyrolysis time has a less significant effect on the physicochemical properties of biochar.

The average yields of JB are 51.99%, 39.82%, 34.08%, 32.60%, and 32.17% at 300, 400, 500, 600, and 700 °C, respectively. As the pyrolysis temperature exceeds 600 °C, the JB yield from jujube biomass changes slightly, which is also indicated by other indicators listed in Table 3. The pH of JB under different carbonization conditions is 7.01–9.83, and the average pH of JB is 8.91, which is similar to those of other lignin-based biochars, but slightly higher than those of cellulose-based biochars [8]. This is mainly because during the pyrolysis of jujube biomass, the acidic substances are pyrolyzed at high temperature and volatilized, while the alkaline substances (alkali salts) are gradually separated from the lignin structure, leading to an increase in the alkaline functional groups, which also form ash. Based on the zeta potential data, the surface of JB is negatively charged, and the amount of negative charge increases with an increase in temperature, implying a stronger electrostatic attraction. The average zeta potential of JB is −47.21 mV. The S_BET_ and pore volume of each JB type are in the 50.92–114.36 m^2^·g^−1^ and 0.10–0.24 cm^3^·g^−1^ range, respectively. The BJH (Barrett–Joyner–Halenda)-adsorption-pore size distribution of JB is shown in Figure S1. According to the definition of the International Union of Pure and Applied Chemistry (IUPAC) [36], the pore structure of JB is mesoporous in this study. It is speculated that the mesoporous structure of JB develops gradually with an increase in the pyrolysis temperature, and a high temperature results in the decreases in the average pore size of JB and thickness of the pore structure, and increases in the number and volume of pores. Therefore, considering these data, 600 °C/2 h can be selected as optimal conditions to obtain biochar. The results obtained herein are in accordance with those published in the literature [8,14].

#### 3.1.2. SEM

**Figure 2:**
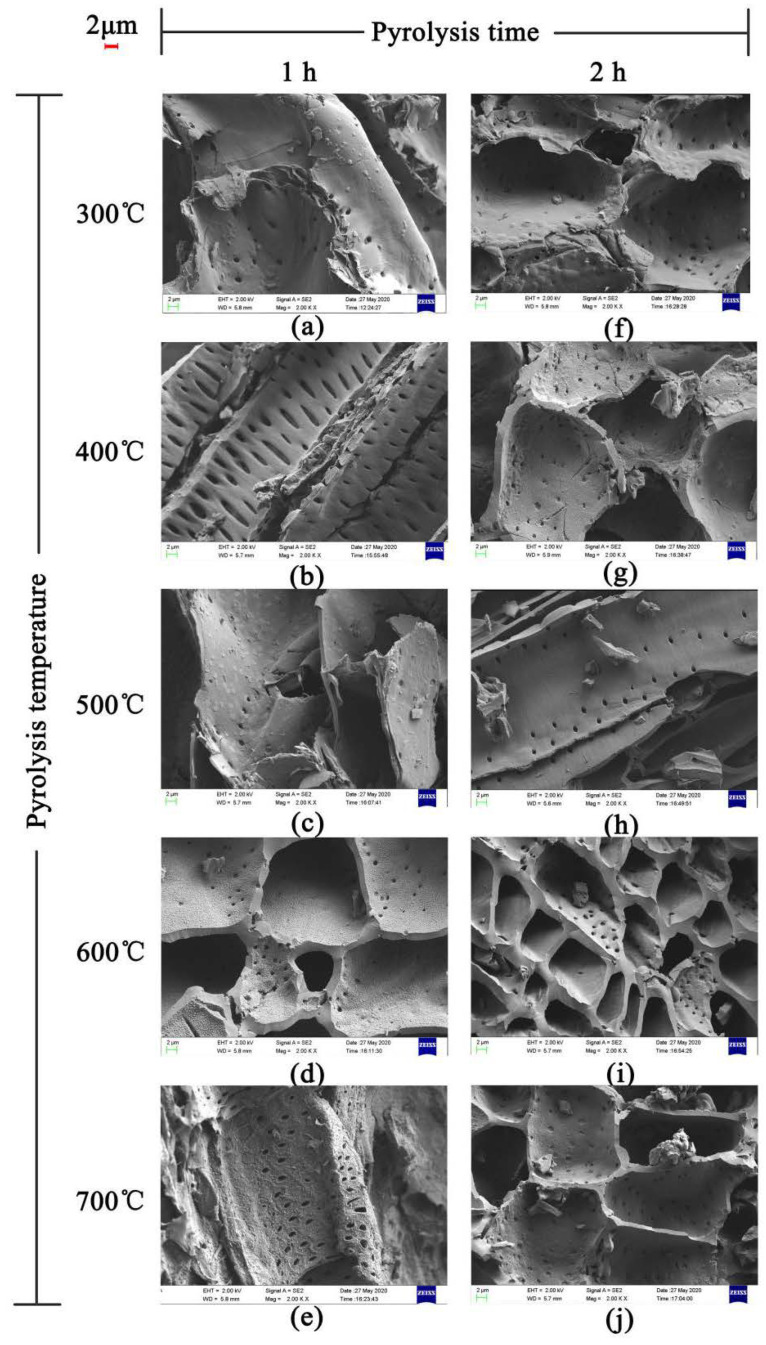
SEM images of JB at 2000 times. (**a**) 300 °C/1 h, (**b**) 400 °C/1 h, (**c**) 500 °C/1 h, (**d**) 600 °C/1 h, (**e**) 700 °C/1 h, (**f**) 300 °C/2 h, (**g**) 400 °C/2 h, (**h**) 500 °C/2 h, (**i**) 600 °C/2 h, (**j**) 700 °C/2 h.

The SEM images of JB are shown in Figure 2. The uneven surface of JB enriched with particles with porous structure can be clearly observed, indicating a squamous texture and random arrangement. The number and size of the pores, as well as the surface texture and particle type vary under different carbonization conditions. As show in Figure 2a–j, with an increase in the pyrolysis temperature, the microscopic morphology of JB shows a gradual development of the microporous structure with a decrease in the thickness of the pore wall and increases in the number of pores and surface roughness. In addition, a more irregular polygonal structure is obtained with some broken particles distributed on the surface of the gradually arranged porous structure as well as the surfaces of some tubular flake structures. A comparison of Figure 2a,f shows that the images are similar, indicating that the effect of pyrolysis time on the porous structure of JB is not as significant as that of the pyrolysis temperature. Furthermore, all SEM images show a clean and smooth biochar surface, which is likely due to the ultrasonication-assisted removal of impurities from the JB surface.

#### 3.1.3. XPS and XRD

**Figure 3:**
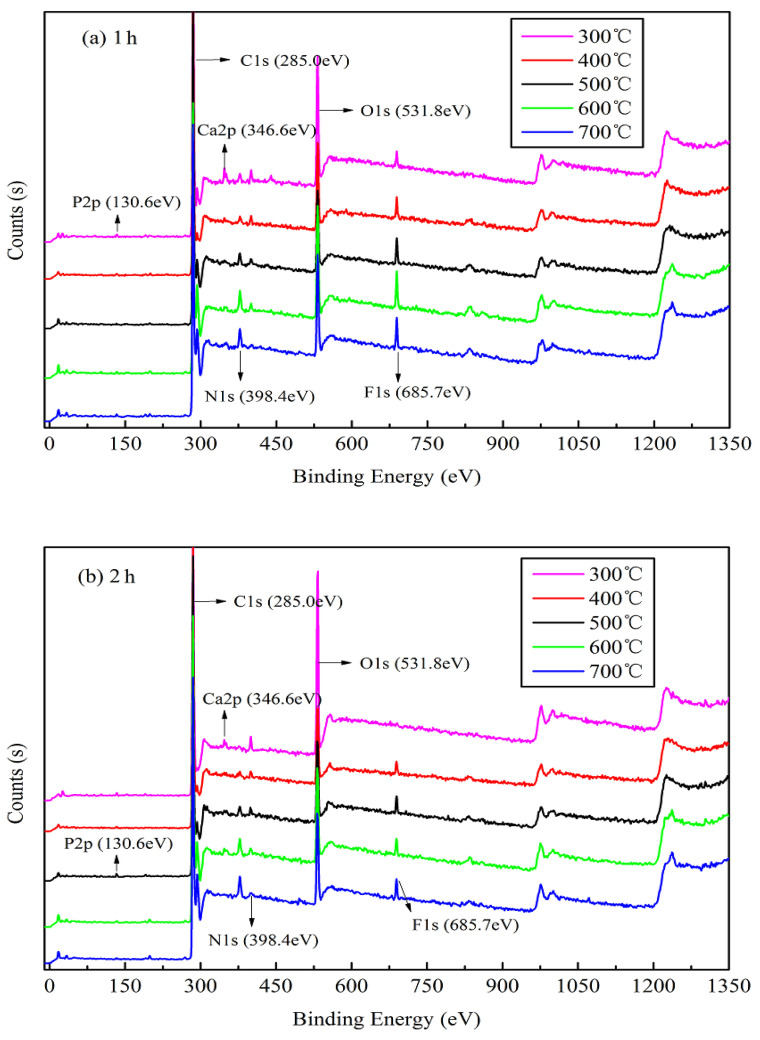
X-ray photoelectron spectra of JB at different carbonization conditions. (**a**) for 1 h; (**b**) for 2 h.

The XPS results shown in Figure 3 indicate the presence of six main elements, C, N, O, F, P, and Ca, upon analysis using a Thermo Avantage v5.979 system (Thermo Fisher Scientific). The binding energies (*E_B_*) of approximately 130.6, 285.0, 346.6, 398.4, 531.8, and 685.7 eV are attributed to P 2 p, C 1 s, Ca 2 p, N 1 s, O 1 s, and F 1 s, respectively. The narrow peak analysis for main elements of XPS as shown in Figure S2. The peaks corresponding to the bonding valence structures of each element slowly appears and tends to be stable as the temperature increases, and the peak intensity becomes higher. As shown in Figure 3a,b, the peaks corresponding to the bonding valence structures of some main elements slowly begin to appear from the start and gradually increase with an increase in the pyrolysis temperature, and their intensities increase with increasing pyrolysis temperature, particularly C 1 s, Ca 2 p, and F 1 s. This suggests that a high pyrolysis temperature can afford a stable elemental morphology and valence-bond structure of jujube biomass during pyrolysis. However, the characteristic peak of O 1 s decreases significantly with an increase in the pyrolysis temperature, which is also confirmed by the elemental analysis (Table 3). This implies that in an oxygen-free environment, jujube biomass can undergo oxidation reactions (combustion) during carbonization as it contains oxygen, which may result in the etching of carbon, thereby promoting the development of pores on the biomass surface to afford a large surface area [37]. 

**Figure 4:**
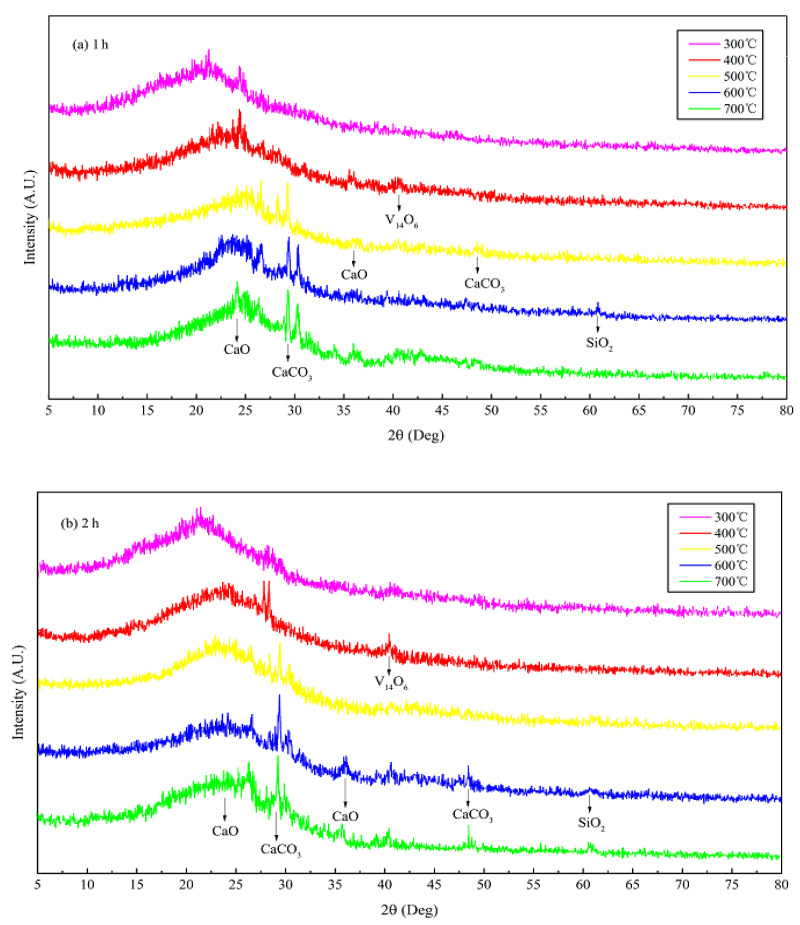
XRD patterns of JB under different carbonization conditions. (**a**) for 1 h; (**b**) for 2 h.

The XRD patterns of JB allow the determination of the mineral composition. Although there is some interference from the noise signals in the XRD data of JB, two characteristic peaks at 26.5° and 29.5° can be roughly observed (Figure 4), indicating the presence of CaO and CaCO_3_, respectively, based on the search of the Jade 6.5 PDF cards (Materials Data Inc., Livermore, CA, USA). The characteristic peaks of CaCO_3_ and CaO become more pronounced with an increase in the pyrolysis temperature, indicating that pyrolysis facilitates crystallization. This also indirectly implies that pyrolysis gradually degrades cellulose, hemicellulose, and lignin in the biomass, releasing ash. The ashes contain alkali salts, mainly calcium carbonate, which result in an alkaline pH of the biochar. This is consistent with the pH values listed in Table 3 and the Ca 2p XPS data (Figure 3). In addition, Figure 3 and Figure 4 show that the pyrolysis time also slightly affects the XPS and XRD data of JB.

#### 3.1.4. FTIR and Thermogravimetric Analysis

**Figure 5:**
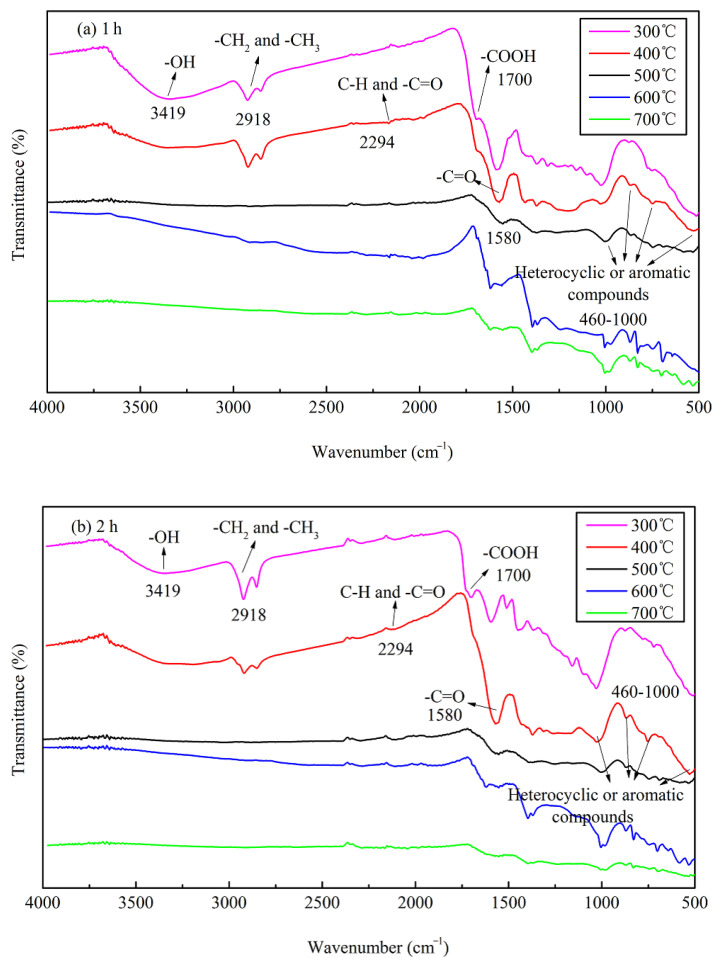
FTIR spectrograms of JB under different carbonization conditions. (**a**) for 1 h; (**b**) for 2 h.

The FTIR data of JB are shown in Figure 5, where six bands for JB are observed at 460–1000, 1200–1400, 1500–1700, 2300, 2900, and 3023–3400 cm^−1^. Based on the data included in “Spectroscopic Tools” website (http://www.science-and-fun.de/tools/) and other reported studies [8,9,11,12,38], the FTIR data of JB shows the presence of -OH group (3419 cm^−1^), methyl and methylene C–H of the alkanes (2918 cm^−1^), C–H and -C=O groups of the fatty acid (2294 cm^−1^), -COOH of the aromatic acids (1700 cm^−1^), amide -C=O (1580 cm^−1^), and pyridine and indole rings of the aromatic compounds (460–1000 cm^−1^), indicating the presence of rich functional groups on its surface. Qualitatively, the position and shape of each peak are similar, indicating that the functional group types of JB prepared under different carbonization conditions are similar. However, some differences are observed in the numbers of these functional groups. The total content of JB surface functional groups decreases with an increase in the pyrolysis temperature. This comprises (1) a decrease in the acidic functional groups including -OH (3419 cm^−1^), methylene (2918 cm^−1^), -COOH (1700 cm^−1^) and -C=O (1580 cm^−1^) groups, (2) increase in the basic functional groups including fatty acid C-H group (2294 cm^−1^), and (3) an increase of the aromaticity in the aromatic compounds (460–1000 cm^−1^). In addition, the pyrolysis time has negligible effect on the type of JB surface functional groups in comparison to that of the ^−1^pyrolysis temperature, which has also been reported in several prior studies [8,39].

**Figure 6:**
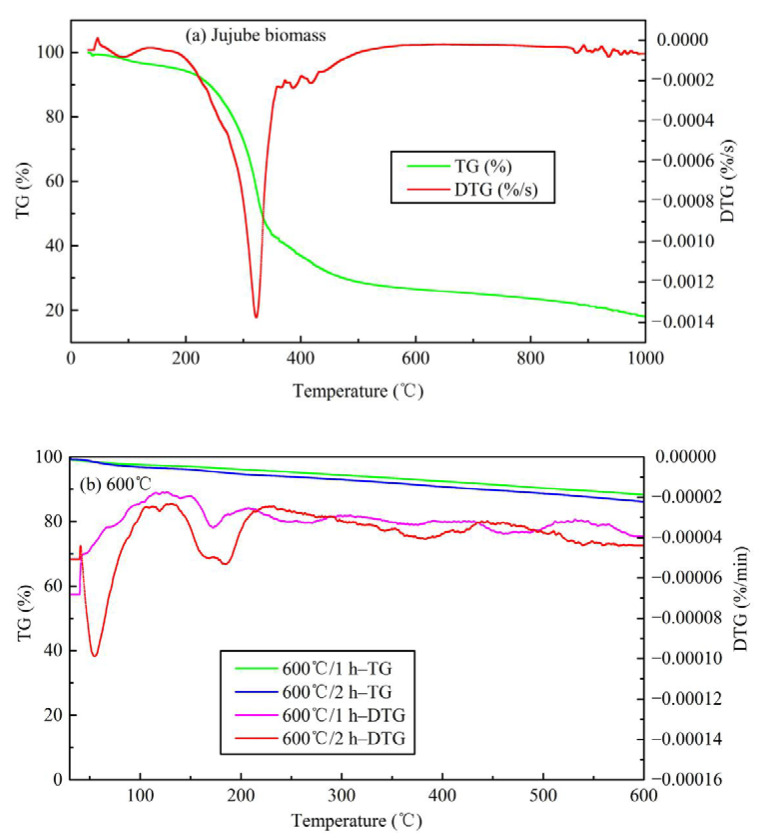
Thermogravimetric analysis of TG and DTG curves for jujube biomass and JB. (**a**) for jujube biomass; (**b**) for 600 °C.

The TG analysis is illustrated in Figure 6. Thermogravimetric analysis of other temperatures is shown in Figure S3. It is evident from Figure 6a that there are four obvious weight loss peaks in the jujube biomass at 80–110 °C, 320 °C, 400–500 °C, and >850 °C, respectively. The first weight loss peak indicated the dehydration of biomass and the volatilization of a small number of substances. The maximum weight loss temperature is about 100 °C, which coincides with the completion of water evaporation. The second weight loss peak is mainly the gradual degradation stage of hemicellulose, cellulose, and lignin, with a maximum weight loss temperature of about 320 °C. The third weight loss peak represents the degradation of lignin which is thermally stable, and the heat loss rate in this process is relatively low. When the temperature is higher than 600 °C, the change of TG and derivative thermogravimetric (DTG) curves tended to be flat and almost straight, indicating that the jujube biomass was completely pyrolyzed and the structure was rearranged, and the type of material was dominated by messy amorphous carbon. Finally, the weight loss stage of biochar occurs at temperatures >800 °C. Some studies reported the crystallization of mineral components and the formation of highly aromatic structures of organic matter in biochar at high temperatures >700 °C [11,21,29], and also the formation of graphite microcrystalline structures with irregular arrangement. If further processed at high temperature, a stable macromolecular fused ring aromatic structure can be formed, which is beneficial to the development of graphitization. Overall, the TG of the jujube biomass has a weight loss of 81.95%, and 18.05% of the remaining mass is mostly fixed carbon and ash, which coincides with the results of the proximate analysis (16.93%). The detailed pyrolysis mechanism is in the discussion section. Taking the pyrolysis temperature of 600 °C as an example, the analysis of the TG loss of JB is shown in Figure 6b. It can be seen that as the pyrolysis temperature continues to rise, the range of DTG axes is small, indicating that the TG loss rate is very low. The weight loss peaks mainly appear at 40–80 °C and 150–200 °C, which may be caused by the absorption of moisture in the air and its volatile ash. When the temperature exceeds 250 °C, the change of TG and DTG curves tended to be flat and almost straight, indicating that the properties of biochar are stable and difficult to change.

### 3.2. Adsorption Application

#### 3.2.1. Optimal Carbonization Parameters

**Figure 7:**
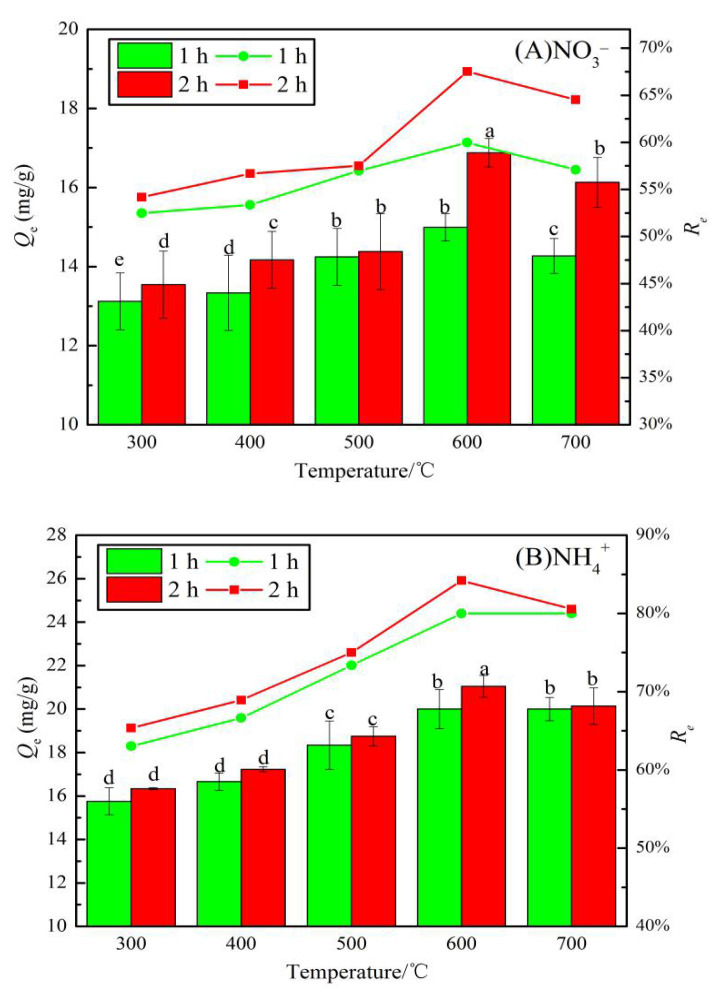
Effects of different carbonization parameters for adsorption capacity and removal rate on JB. (**A**) for NO_3_^−^, (**B**) for NH_4_^+^. Note: a, b, c, and d in the figure are the output results of one-way Analysis of Variance (ANOVA) at P < 0.05 for each treatment.

Figure 7 shows the adsorption capacities and removal rates of NO_3_^−^ and NH_4_^+^ by JB using different carbonization parameters. A comparison of Figure 7A,B shows that the *Q_e_* and *R_e_* values of NO_3_^−^ and NH_4_^+^ by JB show an upward trend followed by a slightly downward trend with an increase in the pyrolysis temperature. However, with an increase in the pyrolysis time, both NO_3_^−^ and NH_4_^+^ show a consistently increasing trend. As shown in Figure 7A, the *Q_e_* value for NO_3_^−^ adsorption by JB at 600 °C for 2 h is the highest (16.88 mg·g^−1^), and the *R_e_* value at this point is 68% (initial concentration of 50 mg·L^−1^). A similar trend is observed in Figure 7B, where at a pyrolysis temperature of 600 °C and time of 2 h, the maximum *Q_e_* and *R_e_* values for NH_4_^+^ adsorption by JB are 21.05 mg·g^−1^ and 84%, respectively. Statistically, the average adsorption capacity for NO_3_^−^ by JB is 14.51 mg·g^−1^ with the corresponding average removal rate of 58%. The average *Q_e_* and *R_e_* values are 18.43 mg·g^−1^ and 74%, respectively, for the NH_4_^+^ adsorption on JB. This suggests that JB shows better adsorption of NH_4_^+^ than NO_3_^−^, and the pyrolysis of the biomass is a suitable method to produce useful carbon materials, where the carbonization parameters significantly affect the *Q_e_* and *R_e_* values of the biochar. This adsorption trend is mainly caused by the specific surface area and the number of surface functional groups [23]. From the above analysis, it can be found that as the temperature increases, the specific surface area increases, while the total surface functional group content decreases, and many functional groups are pyrolyzed away at 700 °C. It is most probably at its best at 600 °C, when it has a larger specific surface area and abundant surface functional groups. Therefore, the carbonization of JB at 600 °C for 2 h is optimal to obtain the biochar type that is used in the following adsorption experiments. 

#### 3.2.2. Addition Dosage

**Figure 8:**
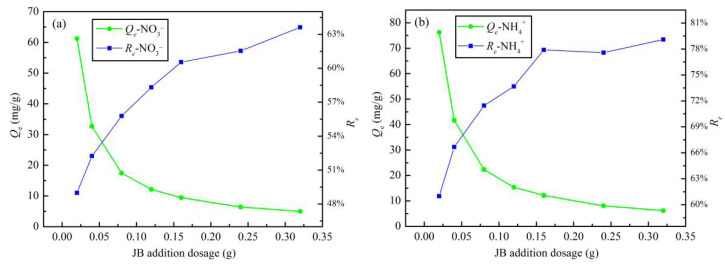
Effects of addition dosage on JB adsorption capacity and removal rate. (**a**) for NO_3_^−^, (**b**) for NH_4_^+^.

The effect of addition dosage on the JB adsorption of NO_3_^−^ and NH_4_^+^ is shown in Figure 8. With an increase in the JB content, the adsorption capacity increases and the removal rates of both NO_3_^−^ and NH_4_^+^ decrease gradually. This can be explained by a mathematical relationship. According to Equations (1) and (2), *R*_e_ = *m* × *Q*_e_/(*V* × *C*_0_), where *C*_0_, and *V* are fixed values. With an increase in the addition dosage, a simple inverse relationship between *Q_e_* and *R*_e_ is followed. This may be related to the functional groups and effective adsorption sites of JB, particularly, the solubility of the adsorbent and the electrostatic induction and repulsion between the adsorption sites [31]. Based on the linear planning from operations research theory, the intersection of the two curves of the adsorption capacity and removal rate is the point where the highest removal rate is obtained at the lowest cost. Thus, an important indicator—i.e., the optimum addition dosage—can be calculated. The optimal dosage of JB for both NO_3_^−^ and NH_4_^+^ adsorption is 1.2 g·L^−1^ (0.06 g/50 mL).

### 3.3. Isotherms Adsorption

**Figure 9:**
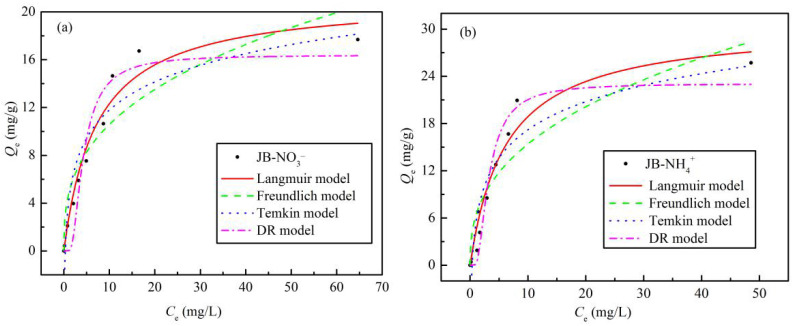
Isotherms adsorption models fitting curves of JB. (**a**) for NO_3_^−^, (**b**) for NH_4_^+^.

The obtained experimental equilibrium data and fitting parameters for NO_3_^−^ and NH_4_^+^ adsorption on JB are shown in Table 4 and Figure 9. With an increase in the initial concentrations of NO_3_^−^ and NH_4_^+^, *Q_e_* increases gradually until saturation is reached. This may be because the initial concentration of the NO_3_^−^ or NH_4_^+^ solution provides a driving force to overcome the mass transfer resistance between the liquid and solid phases, such that an the increase in the initial solution concentration is beneficial to improve the adsorption of biochar [32]. In addition, the Langmuir model affords the best isotherm fittings for both NO_3_^−^ and NH_4_^+^ adsorption on JB, exhibiting the highest fitting coefficient, *R*^2^. This result implies that the adsorption of NO_3_^−^ and NH_4_^+^ on JB is similar to monolayer adsorption and is dominated by physical adsorption [8,33]. As shown in Table 4, the theoretical maximum adsorption capacities for NO_3_^−^ and NH_4_^+^ in the Langmuir model are 21.17 and 30.57 mg·g^−1^, respectively. The *Q_m_* value is very close to the obtained experimental value, which also indirectly indicates that the Langmuir model can appropriately describe the isothermal process. The *R*_L_ values of NO_3_^−^ and NH_4_^+^ adsorption are between 0 and 1, thus showing that these adsorption processes are beneficial and can be carried out smoothly. Based on the physical significance of the Freundlich, Temkin, and D-R model parameters as well as our previously published results in this area [8], the NO_3_^−^ and NH_4_^+^ adsorption on JB is steady, and can also be affected by chemisorption, probably due to ion exchange. 

### 3.4. Adsorption Kinetics

**Figure 10:**
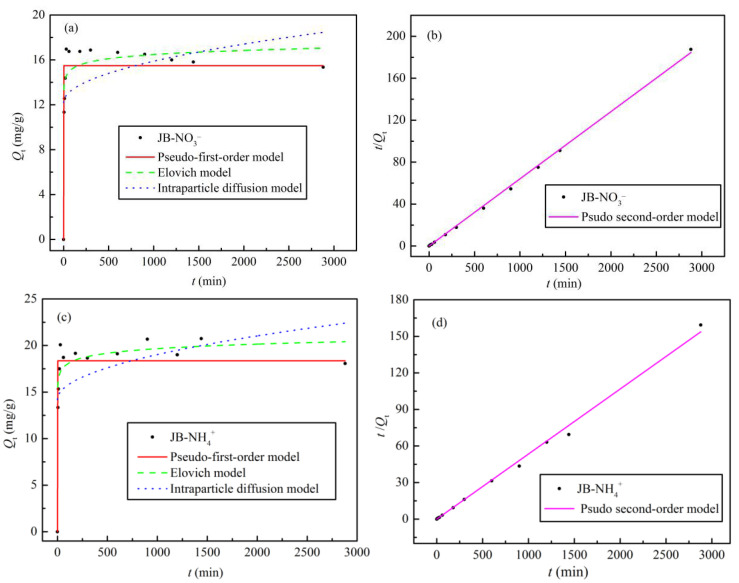
Adsorption kinetics models fitting curves of JB. (**a**,**b**) for NO_3_^−^ at different models, (**c**,**d**) for NH_4_^+^ at different models.

The adsorption kinetic data for NO_3_^−^ and NH_4_^+^ adsorption on JB were analyzed using four models, and the fitted parameters and curves are shown in Table 5 and Figure 10, respectively. With an increase in the adsorption time, the *Q_e_* values for NO_3_^−^ and NH_4_^+^ first increase rapidly, followed by slight fluctuation of the curves, with adsorption equilibrium reached at 60 min (Figure 10a,c). As shown in Table 5 and Figure 10b,d, the pseudo-second-order model affords the highest correlation coefficient (R^2^ > 0.99) of all the models. The results indicate that NO_3_^−^ and NH_4_^+^ adsorption on JB is a composite reaction including intraparticle diffusion, external liquid film diffusion, and surface adsorption [34], thereby suggesting that it is a physical–chemical process. Furthermore, based on the physical significance of the parameters of the pseudo-first-order, Elovich, and intraparticle diffusion models, it is found that the model coefficient, adsorption capacity *Q_e_*, initial adsorption rate *h*, sorption ratio *a*, and boundary layer influence constant *C* of JB are all greater for NH_4_^+^ compared to those for NO_3_^−^. However, JB has a lower desorption constant *b* for NH_4_^+^ than that for NO_3_^−^. These results imply that NH_4_^+^ adsorption on JB is significantly better than that of NO_3_^−^, which is mainly owing to the different adsorption mechanisms.

### 3.5. Pyrolysis Mechanisms and Adsorption Mechanisms

A biomass is typically composed of complex polymeric organic polymers such as cellulose, hemicellulose, and lignin, and the biomass pyrolysis includes the pyrolysis of these three main components. According to Aykac et al. [1], jujube biomass contains 64.42% of holocellulose, of which α-cellulose accounts for 34.45%, 35.75% of lignin, and 1.82% of water and impurities. Unfortunately, there are limited studies reported in the literature describing the pyrolysis mechanism, and based on the results reported by Fakayode et al. [35] and Wang et al. [8], it is assumed that the possible pyrolysis mechanism of jujube biomass consists of three stages. In the first stage, water in the biomass is evaporated at 105 °C. With an increase in temperature in the second stage, holocellulose begins to undergo pyrolysis, mainly with hemicellulose softening at 200–295 °C and cellulose decomposition at 240–375 °C, producing volatile components as the main products and some biochar. The volatile components are mostly acidic, and some acidic or oxygenated functional groups are lost at this stage [8]. In addition, the biochar walls become thin with a decrease in the diameters of the fibers and the surface shows some porosity, which is associated with the rapid accumulation and sustained release of pyrolytic gas. In the third stage at 280–500 °C, lignin decomposition occurs to mainly produce biochar and some volatile components. The pore structure of biochar tends to become more complex and homogeneous with the rapid aggregation and precipitation of the volatile fraction, thereby increasing the gas products, and the simple pore structure becomes dense and diverse, forming numerous mesopores around and inside the large pores. The alkaline functional groups and aromaticity of biochar increase at this stage. The continuous increase of volatile matter has made the pore structure of biochar more complicated and uniform. It is reflected in the simple pore structure becoming dense and diverse, and a large number of mesopores are formed around and inside the larger pores. These speculations are corroborated by the characterization of the physicochemical properties, particularly, the changes in the elemental contents, pH, S_BET_ (Table 3) and SEM data (Figure 2).

**Figure 11:**
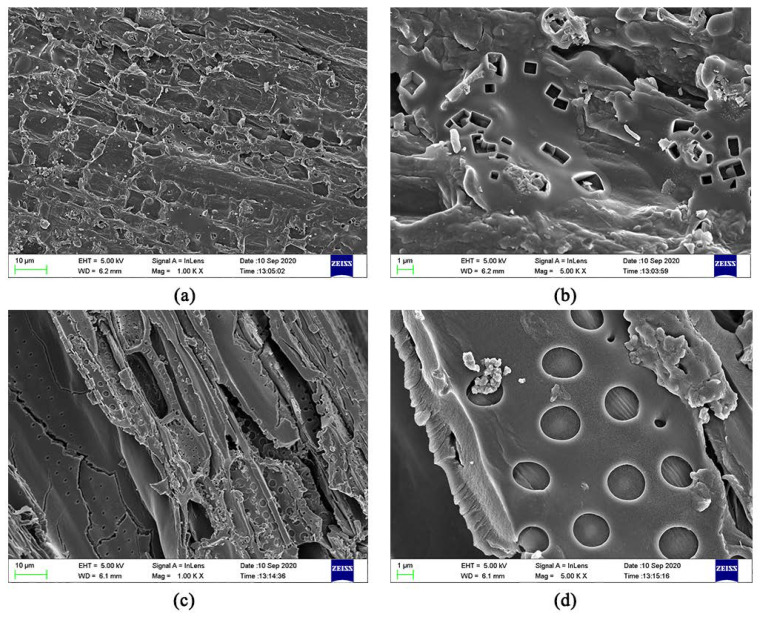
SEM images of JB after adsorption. (**a**) for adsorption of NO_3_^−^ at 1000 times; (**b**) for adsorption of NO_3_^−^ at 5000 times; (**c**) for adsorption of NH_4_^+^ at 1000 times; (**d**) for adsorption of NH_4_^+^ at 5000 times.

**Figure 12:**
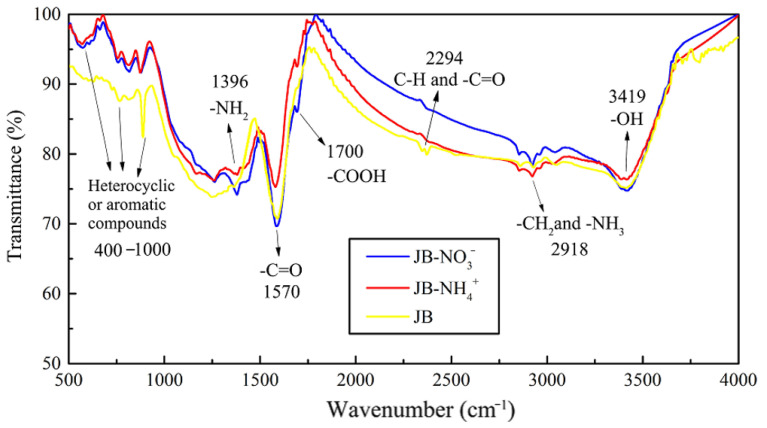
FTIR spectrograms of JB and after the absorption of NO_3_^−^ and NH_4_^+^.

**Figure 13:**
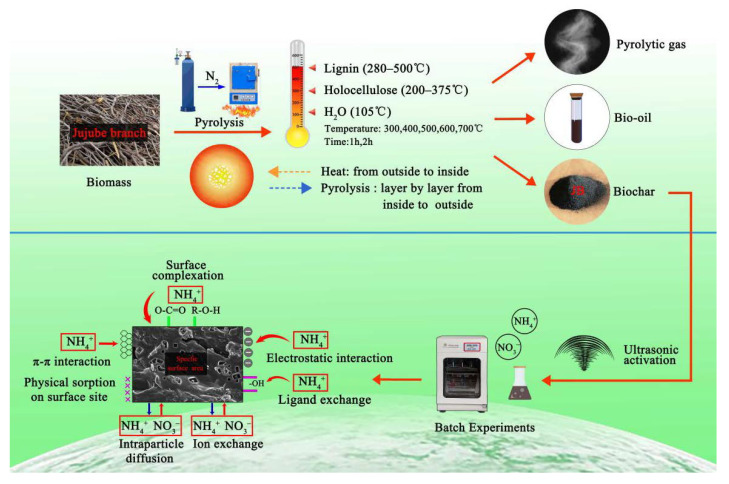
Pyrolysis and adsorption mechanisms of JB for NO_3_^−^ and NH_4_^+^.

Based on the results described in Section 3.3 and Section 3.4 in this study, the best isothermal and kinetic adsorption models for NO_3_^−^ and NH_4_^+^ adsorption on JB are the Langmuir and pseudo-second-order models, respectively. This suggests that the adsorption mechanism is a physicochemical process, which is dominated by physical interactions. The physical adsorption is mainly owing to the mesoporous structure, specific surface area [14,40], and polarity (negative surface charge) of JB, which can not only provide adsorption sites but also afford adsorption via electrostatic attraction. Therefore, the physical adsorption mainly comprises surface adsorption (contact adsorption, similar to gravitational interaction between the mass objects), intraparticle diffusion, and electrostatic interaction for NO_3_^−^ and NH_4_^+^ adsorption on JB. This can be clearly observed in the SEM data collected after adsorption (Figure 11), and is widely accepted by the scholars [14,41,42]. As shown in Figure 11, the pores of the JB surface are filled with adsorbed NO_3_^−^ and NH_4_^+^, and a white residue is also present inside the pores. Furthermore, chemisorption is confirmed by the FTIR data of JB after NO_3_^−^ and NH_4_^+^ adsorption (Figure 12). For NO_3_^−^ adsorption on JB, the FTIR spectra of JB and JB-NO_3_^−^ are similar, but the peaks for -OH at 3419 cm^−1^ and -COOH at 1700 cm^−1^ may be subject to ion-exchange phenomena with NO_3_^−^ because of the negative charges of these two functional groups. For NH_4_^+^ adsorption on JB, the two FTIR spectra are different. As shown in Figure 12, the vibrational peaks of -OH at 3419 cm^−1^, carboxyl group at 1700 cm^−1^, and -C=O at 1570 cm^−1^ change, implying that NH_4_^+^ undergoes ligand exchange or surface complexation, as these functional groups are negatively charged and facilitate cation adsorption. The vibrational peaks of the aromatic heterocyclic compounds at 460–1000 cm^−1^ are also more pronounced, which may imply that the π electrons provided by the aromatic heterocyclic compounds form a stable structure with NH_4_^+^. Moreover, NH_4_^+^ may be ion-exchanged with an amino group (-NH_2_) at 1396 cm^−1^. The adsorption mechanism is included in Figure 13.

### 3.6. Comparison of Adsorption Capacity

Table 6 summarizes the data for NO_3_^−^ and NH_4_^+^ adsorption using different biochars. JB adsorbs relatively higher amounts of NO_3_^−^ and NH_4_^+^ compared to the biochars derived from other biomass feedstocks. The isotherms and kinetics analysis clearly show that the maximum adsorption amount, capacity, strength, stability (low desorption rate), and rate of JB for NH_4_^+^ adsorption are higher than those for NO_3_^−^ adsorption. This indicates that JB is more suitable for the removal of NH_4_^+^ under appropriate conditions. Considering the cost of biochar preparation and good adsorption performance after physical activation, JB can be further developed for usage in practical applications without requiring complex chemical modifications. In addition, it is roughly calculated that the cost of producing one ton of JB is about $100, which is worthy of further development and promotion due to its low cost and efficient adsorption performance for nitrogenous wastewater.

## 4. Conclusions

This paper investigates a new insight to the integrated use of jujube biomass resources, a medium-temperature pyrolysis process to obtain biochar. Overall, the pyrolysis temperature is a more important carbonization parameter than pyrolysis time. As the pyrolysis temperature increases from 300 °C to 700 °C, the content of C element, pH, S_BET_, and the alkaline functional groups of JB continued to increase, while the content of O and N element, yield, Zeta potential, and the total functional groups of JB gradually decreased. The high temperature makes the biochar more stable and has a uniform mesoporous structure. In adsorption applications, the activated JB exhibited the highest adsorption capacity of NO_3_^−^ and NH_4_^+^ at 600 °C for 2 h. The maximum adsorption capacity by the Langmuir model for NO_3_^−^ and NH_4_^+^ reached 21.17 and 30.57 mg·g^−1^, respectively. Therefore, the JB from jujube biomass could be used as an innovative product to provide new ideas for the world of jujube industry.

## Figures and Tables

**Table 1:** List of isotherms and kinetics models.

Models	Expression	Parameters
Langmuir	$Q_{e}=\frac{aQ_{m}C_{e}}{1+aC_{e}}$, $R_{L}=\frac{1}{1+aC_{0}}$	*Q_m_*, *a*
Freundlich	$Q_{e}=K_{F}{C_{e}}^{1/n}$	*K_F_*, 1/*n*
Temkin	$Q_{e}=B\ln A+B\ln C_{e}$	*A*, *B*
Dubinin–Radushkevich (D-R) model	$\ln Q_{e}=\ln Q_{0}-\beta \varepsilon^{2}$, $\varepsilon =RT\ln(1+\frac{1}{C_{e}})$ $E=\frac{1}{{\left(2\beta \right)}^{0.5}}$	*Q*_0_, *β*, *ε,E*
Pseudo-first-order	$Q_{t}=Q_{e}(1-\exp(-\frac{k_{1}}{2.303}t))$	*Q*_e_, *k*_1_
Pseudo-second-order	$Q_{t}=\frac{k_{2}{Q_{e}}^{2}t^{\;}}{1+k_{2}Q_{e}t}$,h=$k_{2}{Q_{e}}^{2}$	*Q*_e_, *k*_2_, *h*
Elovich model	$Q_{t}=\frac{1}{b}\ln(a^{\prime }bt+1)$	*a’*, *b*
Intraparticle diffusion	$Q_{t}=k_{i}t^{0.5}+C$	*C*, *k_i_*

**Table 2:** Proximate analysis of jujube biomass using the dry base.

Sample	Moisture (%)	Volatile Organic Matter (%)	Fixed Carbon (%)	Ash (%)
Jujube biomass	8.25	74.83	16.12	0.81

**Table 3:** Basic physicochemical properties of JB produced from jujube using the dry base.

JB	*C*(%)	*O*(%)	*N*(%)	*O/C*		(*O* + *N*)/*C*		Yield (%)	pH	Zeta	S_BET_	Pore Volume (cm^3^·g^−1^) ^2^	Average Pore Size (nm) ^3^
				Value	Uncertainty ^1^	Value	Uncertainty			(mV)	(m^2^·g^−1^)		
300 °C/1 h	67.86	20.84	2.73	0.31	0.43	0.35	0.49	50.18	7.01	−41.5	50.92	0.10	49.42
400 °C/1 h	70.20	18.03	1.75	0.26	0.36	0.28	0.40	40.75	8.22	−48.1	72.77	0.13	46.43
500 °C/1 h	72.02	14.81	1.81	0.21	0.29	0.23	0.33	34.48	9.79	−47.3	83.05	0.15	32.67
600 °C/1 h	75.64	15.67	1.41	0.21	0.29	0.23	0.32	32.53	9.78	−52.4	88.23	0.15	14.43
700 °C/1 h	78.64	14.77	1.37	0.19	0.27	0.21	0.29	31.64	9.83	−50.5	99.62	0.19	8.57
300 °C/2 h	69.60	20.96	3.1	0.3	0.43	0.35	0.49	53.79	7.52	−42.2	51.61	0.09	31.43
400 °C/2 h	73.09	19.96	1.79	0.27	0.39	0.3	0.42	38.91	8.53	−51.4	76.4	0.12	17.91
500 °C/2 h	76.41	14.77	2.41	0.19	0.27	0.22	0.32	33.67	9.1	−45.0	82.2	0.17	11.26
600 °C/2 h	77.88	14.07	1.97	0.18	0.26	0.21	0.29	32.67	9.61	−49.6	103.82	0.21	8.78
700 °C/2 h	77.92	15.01	1.83	0.19	0.27	0.22	0.31	32.69	9.73	−44.1	114.36	0.24	7.02

^1^ Using Gaussian propagation to measure the uncertainty of elemental analysis. ^2^ Single point adsorption total pore volume. ^3^ Total adsorption average pore width (4 V/S_BET_).

**Table 4:** Isotherm model parameters of NO_3_^−^ and NH_4_^+^ by JB.

Adsorption Type	Langmuir				Freundlich			Temkin			D-R Model		
	*K_L_*	*Q_m_*/mg·g^−1^	*R_L_*	*R* ^2^	*K*_F_	*n*	*R* ^2^	*A*	*B*	*R* ^2^	*Q*_0_/mmol·g^−1^	*E*/kJ·mol^−1^	*R* ^2^
NO_3_^−^	0.14	21.17	0.07~0.88	0.96	4.68	2.83	0.85	3.18	3.41	0.88	16.40	0.43	0.72
NH_4_^+^	0.16	30.57	0.06~0.86	0.95	6.32	2.58	0.83	2.83	5.15	0.86	23.11	0.55	0.78

**Table 5:** Kinetic model parameters of NO_3_^−^ and NH_4_^+^ by JB.

Adsorption Type	Pseudo-First-Order			Pseudo-Second-Order				Elovich			Intraparticle Diffusion		
	*Q_e_*/mg·g^−1^	*k*_1_	*R* ^2^	*Q_e_*/mg·g^−1^	*k*_2_	*h/*mg·g^−1^·min^−1^	*R* ^2^	*a*/g·mg^−1^·min^−1^	*b/*g·mg^−1^	*R* ^2^	*k_i_*	*C*	*R* ^2^
NO_3_^−^	15.49	2.13	0.84	15.60	0.01	2.43	0.99	1.37	1.87	0.90	0.11	12.20	0.10
NH_4_^+^	18.36	1.82	0.85	18.75	0.01	3.52	0.99	9.17	1.42	0.91	0.15	14.19	0.15

**Table 6:** Comparison of the adsorption capacity onto various biochars derived from different materials.

Raw Materials	Adsorption Type	Maximum Theoretical Adsorption Capacity/mg·g^−1^	Literature
Date palm	NO_3_^−^	8.37	[29]
Rice husk	NO_3_^−^	2.1	[43]
Corn stover	NO_3_^−^	6.25	[44]
Switchgrass	NO_3_^−^	1.84	[44]
Ponderosa pine wood	NO_3_^−^	6.20	[44]
Jujube	NO_3_^−^	21.10	This work
*P. australis*	NH_4_^+^	2.82	[30]
*C. indica*	NH_4_^+^	13.35	[30]
*Z. caduci* *flora*	NH_4_^+^	2.41	[30]
*T. dealbata*	NH_4_^+^	4.93	[30]
*P. purpureum Schum*	NH_4_^+^	7.36	[30]
Jujube	NH_4_^+^	30.57	This work

## Supplementary Materials

The following are available online at https://www.mdpi.com/1996-1944/13/24/5594/s1. Table S1. Supplementary elemental analysis of JB using the dry base; Figure S1. BJH-adsorption-pore size distribution of JB at 600 °C/2 h. Figure S2. X-ray photoelectron spectra of JB at 600 °C/2 h. (a) for C 1 s; (b) for N 1 s; (c) for O 1 s; (d) for Ca 2 p. Figure S3. Thermogravimetric analysis of TG and DTG curves for jujube biomass and JB. (a) for 300 °C; (b) for 400 °C; (c) for 500 °C; (d) for 700°C.
