# Supplementary material for: Utilization of Jujube Biomass to Prepare Biochar by Pyrolysis and Activation: Characterization, Adsorption Characteristics, and Mechanisms for Nitrogen
Source: Materials (Basel). 2020 Dec 8;13(24):5594. doi: 10.3390/ma13245594 (PMC7764758; doi:10.3390/ma13245594)
Supplement: Supplementary file 1 [file materials-13-05594-s001.pdf]

materials

MDPI

Supplementary Materials

Utilization of Jujube Biomass to Prepare Biochar by Pyrolysis and Activation: Characterization, Adsorption Characteristics, and Mechanisms for Nitrogen

Di Zhang [1,†], Tongtong Wang [2,†], Jinhu Zhi [1,3,*], Qiangqing Zheng [4], Qiling Chen [4], Cong Zhang [1,3], and Yalong Li [5]

1 College of Plant Sciences, Tarim University, Alar 843300, China; dizhang@nwafu.edu.cn (D.Z.); zc1569325100@163.com (C.Z.)
2 College of Natural Resources and Environment, Northwest A & F University, Yangling 712100, China; wangtongtong1993@nwafu.edu.cn
3 Research Center of Oasis Agricultural Resources and Environment in Sourthern Xinjang, Tarim University, Alar 843300, China
4 Institute of forestry and horticulture, Xinjiang Academy of Agricultural Reclamation Sciences, Shihezi 832000, China; zhengqq369@163.com (Q.Z.); cql619@163.com (Q.C.)
5 Changjiang River Scientific Research Institute, Wuhan 430012, China; lyalong888@163.com
* Correspondence: zjhzky@163.com; Tel.: +86-136-5759-3850
† These authors contributed equally to this paper.

Received: 12 November 2020; Accepted: 4 December 2020; Published: 8 December 2020

1. Characterisation

The C, O, and N contents were measured using a Vario EL cube elemental analyser (Elementar Analysensysteme GmbH, Berlin, Germany) with argon as the carrier gas. Based on this measurement method, the element content of H (percentage content) can be calculated indirectly from the content of the other three elements, that is: "H=100%-C-O-N. The pH was measured using a PHS-3E pH metre (Malvern Panalytical Ltd., Shanghai, China). The yield of the biochar was equal to the ratio of the biochar mass produced via pyrolysis to the mass of the original dried Jujube. The zeta potential was measured using a NanoZS90new zeta potential analyser (Malvern Panalytical Ltd., Malvern, UK), and proximate analysis was performed using a XKGF-6000A automatic industrial analyser (Henan Xinke Analytical Instruments Co., Ltd, Hebi, China). Scanning electron microscopy (SEM) of the JB samples was performed using a Sigma HD scanning electron microscope (Carl Zeiss AG, Oberkochen, Germany) at 2.0 kV, which was equipped with an X-MAXEDS spectroscopic detection system (Oxford Instruments, Oxford, UK). The Brunauer–Emmett–Teller (BET) specific surface area (S_{BET}) and pore size were determined using N_2 as the adsorbate at 77 K and relative pressure of 0.05–0.20 by employing an ASAP2020 plus analyser (Micromeritics Instruments Co., Norcross, Georgia, GA, USA). All JB samples were scanned using an Escalab 250 Xi X-ray photoelectron spectrometer (Thermo Fisher Scientific, Waltham, Massachusetts, MA, USA) to obtain the X-ray photoelectron spectroscopy (XPS) data. The mineral species of the JB samples were identified by X-ray diffraction (XRD) using a D-MAX 2500 X-ray powder diffractometer (Rigaku Corp., Tokyo, Japan). The measurements were performed by employing a scan step size of 0.02, scan speed of 2 deg·min^{-1}, receiving slit width of 0.15 at 30–40 kV and 30–40 mA. Fourier-transform infrared (FTIR) data were obtained using KBr pellets and a Vertex70 FTIR spectrometer (Bruker Corp., Billerica, Massachusetts, MA, USA) with 16 scans over 400–4000 cm^{-1} at a resolution of 2 cm^{-1}. Thermogravimetric analysis (TGA) was performed using a standard method with a TGA2 analyser (Mettler Toledo, Columbus, Ohio, OH, USA).

2. Data analysis

In this work, data statistics were calculated using Excel 2016 Pro (Microsoft, Redmond, WA, USA) and SPSS 26.0 (IBM Corporation, Armonk, NY, USA). One-way ANOVA was conducted to compare the means of the measured values at $P < 0.05$ for each treatment. Isothermal adsorption and adsorption kinetic curves were fitted and plotted using OriginPro 2018b (OriginLab, Northampton, MA, USA).

Table S1. Supplementary elemental analysis of JB using the dry base.

JB	C(%)	H(%)	O(%)	N(%)	O/C	H/C	(O+N)/C
300℃/1h	67.86	8.57	20.84	2.73	0.31	0.13	0.35
400℃/1h	70.20	10.02	18.03	1.75	0.26	0.14	0.28
500℃/1h	72.02	11.36	14.81	1.81	0.21	0.16	0.23
600℃/1h	75.64	7.28	15.67	1.41	0.21	0.10	0.23
700℃/1h	78.64	5.22	14.77	1.37	0.19	0.07	0.21
300℃/2h	69.60	6.34	20.96	3.10	0.30	0.09	0.35
400℃/2h	73.09	5.16	19.96	1.79	0.27	0.07	0.30
500℃/2h	76.41	6.41	14.77	2.41	0.19	0.08	0.22
600℃/2h	77.88	6.08	14.07	1.97	0.18	0.08	0.21
700℃/2h	77.92	5.24	15.01	1.83	0.19	0.07	0.22

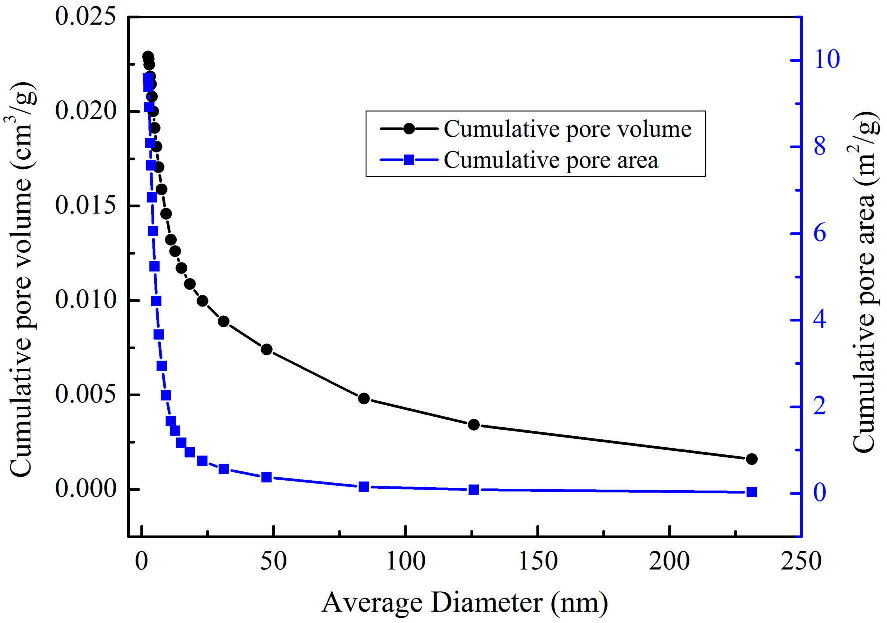

Figure S1. BJH (Barren-Joyner-Halenda)-adsorption-pore size distribution of JB at 600 °C/2h.

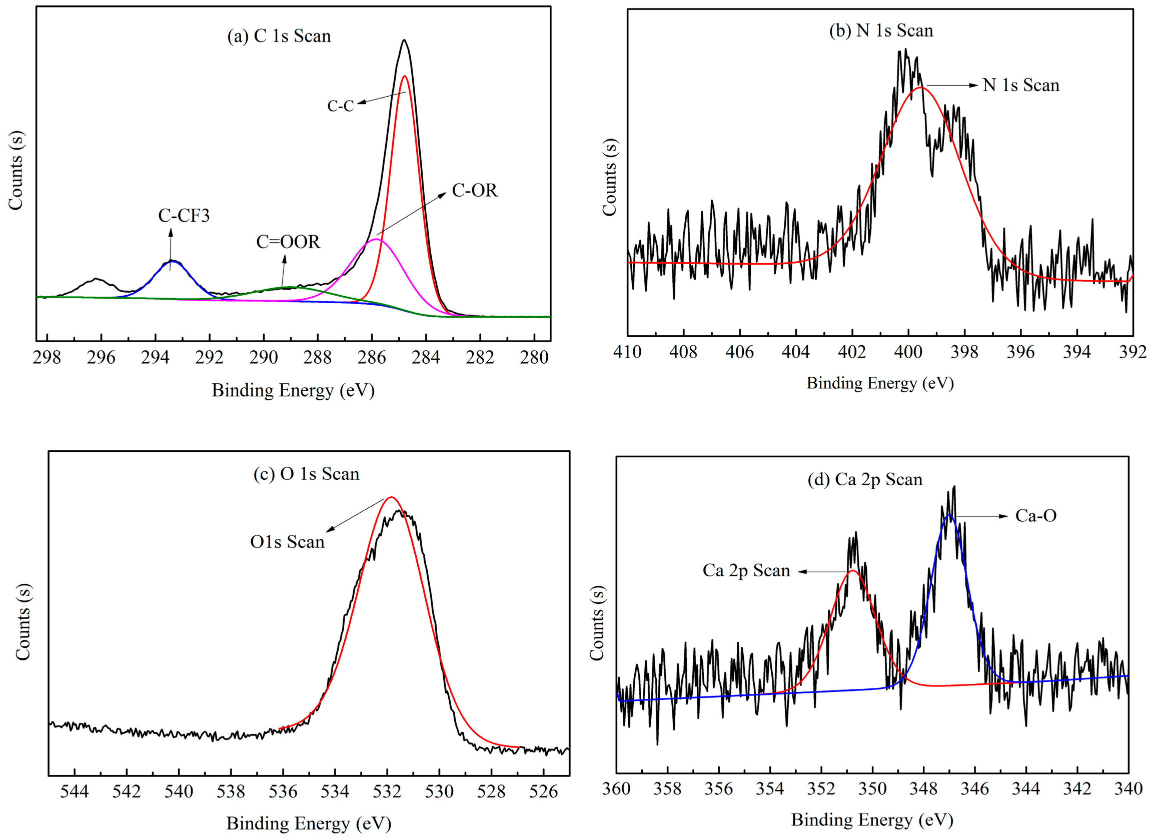

Figure S2. X-ray photoelectron spectra of JB at 600 °C/2h. (**a**) for C 1s; (**b**) for N 1s; (**C**) for O 1s; (**d**) for Ca 2p.

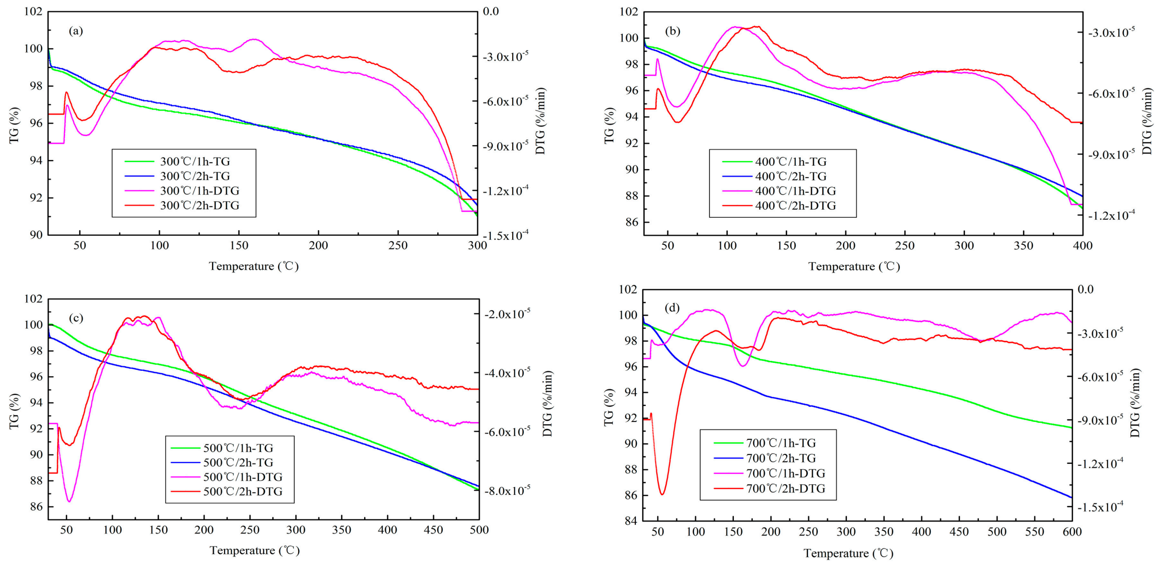

Figure S3. Thermogravimetric analysis of TG and DTG curves for jujube biomass and JB. (**a**) for 300 °C; (**b**) for 400 °C; (**c**) for 500 °C; (**d**) for 700 °C.
